# Recent Advances in the Biocontrol of Nosemosis in Honey Bees (*Apis mellifera* L.)

**DOI:** 10.3390/jof8050424

**Published:** 2022-04-20

**Authors:** Massimo Iorizzo, Francesco Letizia, Sonia Ganassi, Bruno Testa, Sonia Petrarca, Gianluca Albanese, Dalila Di Criscio, Antonio De Cristofaro

**Affiliations:** 1 Department of Agricultural, Environmental and Food Sciences (DiAAA), University of Molise, Via De Sanctis snc, 86100 Campobasso, Italy; iorizzo@unimol.it (M.I.); f.letizia@studenti.unimol.it (F.L.); bruno.testa@unimol.it (B.T.); sonia_petrarca@libero.it (S.P.); d.dicriscio@unimol.it (D.D.C.); decrist@unimol.it (A.D.C.); 2 Conaproa, Consorzio Nazionale Produttori Apistici, 86100 Campobasso, Italy

**Keywords:** beneficial microbes, biocontrol, nosemosis, plant extract, RNAi

## Abstract

Nosemosis is a disease triggered by the single-celled spore-forming fungi *Nosema apis* and *Nosema ceranae*, which can cause extensive colony losses in honey bees (*Apis mellifera* L.). Fumagillin is an effective antibiotic treatment to control nosemosis, but due to its toxicity, it is currently banned in many countries. Accordingly, in the beekeeping sector, there is a strong demand for alternative ecological methods that can be used for the prevention and therapeutic control of nosemosis in honey bee colonies. Numerous studies have shown that plant extracts, RNA interference (RNAi) and beneficial microbes could provide viable non-antibiotic alternatives. In this article, recent scientific advances in the biocontrol of nosemosis are summarized.

## 1. Introduction

The microsporidia *Nosema apis* and *Nosema ceranae* are among the main pathogens of honey bees; they are spore-forming, obligate, intracellular parasites and are acknowledged as belonging to the kingdom of Fungi [1,2].

Most recently, Tokarev et al. [3] placed the *Nosema* species, which infects bees (*Anthophila*, Hymenoptera), under the new genus *Vairimorpha*. *N. apis*. This new genus was first isolated from the European honey bee *Apis mellifera* (*Hymenoptera*, Apidae), whereas *N. ceranae* was first reported from the Asian honey bee *Apis cerana* (*Hymenoptera*, Apidae). Currently, these two parasites have a worldwide distribution [4,5,6,7,8,9,10,11,12,13,14,15,16]. 

Both *N. apis* and *N. ceranae* are the etiological agents of nosemosis, one of the adult honey bee’s most widespread and serious diseases, causing significant economic losses to beekeepers [5,17,18,19]. *N. apis* is responsible for nosemosis type A, a disease that increases bee mortality in winter and causes a slow build-up in spring, making bees weak and reducing honey yield [20]. Field experiments demonstrated that *N. apis* infection is also responsible for the onset of foraging at a younger age than in healthy worker bees. [21,22]. Dosselli et al. [23] demonstrated that *N. apis* infected worker bees quickly altered their flight behavior, reducing the foraging trip duration and increasing the number of flights. In addition, the disease causes diarrhea and fecal spots inside and outside the hive [9]. Nosemosis type C, caused by *N. ceranae* [24], includes a wide range of effects on honey bee physiology and behavioral changes, weakness and colony mortality increase, decreased brood-rearing capacity and honey production, all of which may contribute to colony collapse [25,26,27,28,29,30,31]. Moreover, *N*. *ceranae* infection may lead to the impairment of hormone production and lipid synthesis [32,33], the induction of nutritional and energetic stress [32,34,35,36] and the degeneration of the host’s midgut tissues [37,38]. *N. ceranae* infection can also induce immune system suppression in the host [39,40]. Recently, different authors demonstrated that both *N. apis* and *N. ceranae* inhibit apoptosis in the host cells [41,42,43].

*N*. *ceranae* infection also affects the neurobiology of honey bees by impairing olfactory learning and memory [44] and, on a behavioral level, premature foraging in worker bees [29,34,45], decreased homing ability [46] and weaker flight ability [47].

The acquisition of *Nosema* occurs via the fecal-oral route through the ingestion of spores. In the midgut lumen, the spores extrude a polar filament through which the sporoplasm is transferred into the epithelial cells and merogony begins. Shortly, meronts can either turn into primary spores or mature spores; primary spores transmit the disease to adjacent cells, whereas mature spores are released into the midgut lumen, from which they can pass through the rectum into the feces or remain in the midgut to infect other cells [5,48,49,50,51]. The spores excreted by the host through the feces may then contaminate the nesting environment, comb, floral resources, collected pollen and water [20,26,52]. Beyond horizontal transmission (e.g., via trophallaxis) [53,54], both *N. apis* and *N. ceranae* may be airborne [55] and sexually transmitted [56,57]. Because of the disastrous consequences of *Nosema* infections, there is a strong demand for the management of these pathogens.

The antibiotic fumagillin is a historically accepted treatment for *Nosema* infections [58]. Unfortunately, *Nosema* spp. strains exhibit variable levels of antibiotic resistance [59]. Furthermore, dysbiosis of the gut microbiota caused by antibiotics might increase the susceptibility of honey bees to *N. ceranae* infections [60]. Although the fumagillin degrades quickly in the hive, residues can persist, and their degradation products pose a potential risk to human health [61,62,63]. Consequently, fumagillin is currently banned in many countries, including the European Union [64], due to its genotoxic and tumorigenic properties towards humans and toxicity to bees [65,66]. The inhibition of the enzyme methionine aminopeptidase type 2 (MetAP2) is fumagillin’s proposed mechanism of action against *Nosema* [67]. Therefore, Van den Heever et al. [66] recently screened other MetAP2 (methionine aminopeptidase type 2) antagonist compounds in cage experiments and observed a significant decrease in load of *N. ceranae* [58]. However, given the tight regulation on the use of antibiotics in animal food, precautions should be taken in the development and approval of novel MetAP2 inhibitors for honey bee diseases. 

Currently, several chemical compounds are used for the management of *Nosema* infections. Formic acid and oxalic acid, both used for *Varroa* control, have the ability to control *Nosema* [68,69]. The mode of action of these organic acids against *N. ceranae* is still unknown. Strachecka et al. [70] reported that oxalic acid interfered with the activity of the cuticle proteolytic defense system in *A. mellifera* workers. Indeed, a notable component of resistance is the layer of active cuticle surface proteins that protect the honey bee from pathogen invasion [71,72]. In a study conducted by Genath et al. [73], formic acid treatment was shown to induce an alteration in the proteostasis of the ectoparasite *Varroa destructor*, with significant dysregulation of proteins involved in mitochondrial cellular respiration. Two other molecules, nitroimidazole compounds (metronidazole, tinidazole), completely inhibit the proliferation of *N. ceranae* and constitute promising candidates for the establishment of a new strategy to control *Nosema* [74].

In recent years, several studies have claimed that residues of veterinary treatments have been reported in hive products, which is a public health concern as their ingestion can pose a risk to human health [75,76,77,78]. In particular, it is relevant to note that the commercial formulation of fumagillin consists of the dicyclohexylamine (DCH) salt of fumagillin in a 1:1 stoichiometric ratio with fumagillin. The stability of DCH, along with its genotoxicity and tumorigenicity, renders it a major potential contaminant in hive products for human consumption [63,65]. Therefore, to prevent and treat nosemosis over the years, developing sustainable alternative methods to fumagillin and finding new natural agents active against *Nosema* spp. has increased [79]. In this review, recent scientific advances on some alternative approaches to control nosemosis are discussed.

## 2. Plant Extracts

In recent years, several studies have evaluated plant extracts and organic compounds, reporting their effectiveness for the biocontrol of nosemosis [80]. In Table 1, we list the plant extracts tested and their main effects on nosemosis.

Some scientific investigations have used products already available on the market. In the trial by Cilia et al. [81], the efficacy of two commercial products, ApiHerb^®^ and Api-Bioxal^®^(Chemicals Laif SpA, Padua, Italy), was compared. ApiHerb^®^ is composed of *Allium sativum* and *Cinnamomum zeylanicum* extracts. Instead, Api-Bioxal^®^ is a registered veterinary drug against *Varroa destructor* containing oxalic acid dihydrate. While both treatments lowered the abundance of *N. ceranae*, ApiHerb^®^ also diminished the prevalence of infected bees.

In a study by Shumkova et al. [82], the findings from the application of two plant extracts, NOZEMAT HERB^®^ and NOZEMAT HERB PLUS^®^ (Extract Pharma, Sofia, Bulgaria), are discussed. The accurate quantitative composition of these two herbal supplements is protected from patent law. Specifically, the authors demonstrated that both supplements significantly improved honey bee colony strength and diminished the number of *N. ceranae* spores by 68% in the group treated with NOZEMAT HERB^®^, while in the group treated with NOZEMAT HERB PLUS^®^, a reduction of 60% was found. Charistos et al. [83] showed that using HiveAlive^TM^ (Advance Science Ltd., Galway, Ireland), a mixture of algae extracts, increased colony worker bee population size by 89% and decreased *N. ceranae* spores by 57%; the effect of this treatment is most likely due to the strengthening of the intestinal epithelium, although the authors do not refer to the survival of the honey bee and use colony mortality as one of the parameters to evaluate the strength of the colony [67]. In an investigation using Nozevit^®^ (a natural product based on plant polyphenols), it was observed that this commercial phytopharmacological supplement could improve the health of honey bees by decreasing *Nosema* spores [84,85]. The same product was used by van den Heever et al. [66] in cage trials with negative results, which is why further investigations are required.

The phytotherapeutic product Protofil^®^, rich in flavonoids (rutin and quercetin) and volatile compounds such as eucalyptol (1.8-cineol) and chavicol-methyl-ether, prevents the growth cycle of *N. apis* [86,87], but in the description of this hydroalcoholic extract, the mechanism of action is not specified. Other studies have evaluated the integration of the honey bee diet with vitamins and nitrogen compounds. Dietary supplementation with an amino acid and vitamin complex called “BEEWELL AminoPlus” (Provet, Ankara, Turkey) decreases *N. ceranae* spores and prevents bees from immune suppression by increasing the expression of genes for immune peptides (abaecin, apidaecin, hymenoptaecin, defensin and vitellogenin) [88] However, not always the products advertised as anti-nosemosis supplements have beneficial effects on honey bees infected with *N. ceranae* [67].

According to the study conducted by Botías et al. [89], three therapeutic agents (Nosestat^®^, Phenyl salicylate and Vitafeed Gold^®^) were screened to control *N. ceranae* infection in bee colonies and compared with the use of fumagillin. Nosestat^®^ is a combination of iodine and formic acid and is commercialized for the treatment and prevention of nosemosis in bees. Vitafeed Gold^®^ is a natural extract based on beet extract and molasses. None of the investigated products were effective against *Nosema* under the used experimental conditions. Among the natural products explored hitherto against nosemosis, there is propolis extract: a mixture of resinous substances collected by bees from various plant sources. Of the emerging effective treatments against *N. ceranae*, propolis extract is effective in three of the four bee species (*A. cerana*, *A. mellifera* and *A. florea*) [90,91,92,93,94,95].

As for the use of extracts obtained from different plant sources, many studies have been carried out with highly promising results, which are sometimes comparable to those obtained with fumagillin. Chaimanee et al. [96] demonstrated that plant extracts made from *Annona squamosa*, *Ocimum basilicum*, *Psidium guajava* and *Syzygium jambos* possess a strong anti-microsporidian activity and inhibit the development of *N. ceranae* spores, with similar efficacy to fumagillin. In another recent study conducted by Özkırım et al. [97], the results showed that a mix of herbal extract mixture containing *Rumex acetosella*, *Achillea millefolium*, *Plantago lanceolata*, *Salvia officinalis*, *Thymus vulgaris*, *Rosmarinus officinalis* and *Laurus nobilis* was more effective than fumagillin.

**Table 1:** List of plant species whose extracts, and relative bioactive compounds, are effective against nosemosis.

Plant Species	Extract	Bioactive Compounds	Relevant Reported Effects	Ref.
*Achillea millefolium*	Aqueous	terpenes and terpenoids (artemisia ketone, camphor, linalyl acetate and 1,8-cineole)	Antimicrobial activity, reduction of *Nosema* spores, improvement of honey bee survival.	[97]
*Agastache foeniculum*	Ethanolic	phenolic acids and flavonoids (chlorogenic acid, isoquercitrin, quercetin, vanillin, acacetin, gallic acid, caffeic acid, p-OH cinnamic acid, resveratrol)	Reduction of *Nosema* spores.	[98]
*Allium sativum*	Ethanolic	essential oils	Reduction of *Nosema* spores.	[99]
*Andrographis paniculata*	Aqueous	terpenoids (andrographolide, dehydrographolide)	Reduction of *Nosema* spores; mitigation of gut epithelium degeneration caused by *N. ceranae*.	[100]
*Annona squamosa*	Ethanolic	steroids, terpenes, alkaloids, flavonoids, saponins, phenolic acids	Reduction of *Nosema* spores.	[96]
*Aristotelia chilensis*	Methanolic	phenolic acids, flavonoids (caffeic acid, apigenin and pinocembrin)	Reduction of *N. ceranae* spore loads, improvement of honey bee survival.	[95]
*Artemisia absinthium*	Ethanolic	flavonoids (isoquercitrin, quercetin, rutin)	Antimicrobial and antioxidant activity, reduction of *Nosema* spore loads.	[98,101]
*Artemisia dubia*	Aqueous	benzopyrones, phenolic compounds and quinic acids derivatives (coumarin, chlorogenic acid, 4,5-dicaffaeoylquinic acid)	*In vitro* and *in vivo* anti-nosemosis activity.	[102,103]
*Aster scaber*	Aqueous	benzopyrones, phenolic compounds and quinic acids derivatives (coumarin, chlorogenic acid, 4,5-dicaffaeoylquinic acid)	*In vitro* and *in vivo* anti-nosemosis activity.	[102,103]
*Brassica nigra*	Organic	glucosinolates (glucoerucin, glucoraphanin, sinigrin) and isothiocyanates	*In vivo* and *in vitro* reduction of *N. ceranae* infections, improvement of honey bee survival.	[104]
*Cryptocarya alba*	Aqueous	terpenes and terpenoids (β-phellandrene, α-terpineol, eucalyptol)	Antimicrobial activity and reduction of *Nosema* spores.	[105]
*Cucurbita pepo*	Ethanolic	Essential Oils	Reduction of *Nosema* spores.	[99]
*Eleutherococcus senticosus*	Ethanolic	saponins and flavonoids (eleutheroside B, eleutheroside E and naringenin)	Prophylactic effect in vivo against *Nosema* infections does not affect *Nosema* spores’ viability, improvement of honey bee survival.	[106]
*Eruca sativa*	Hexan	glucosinolates (glucoerucin, glucoraphanin, sinigrin)	*In vivo* and *in vitro* reduction of *N. ceranae* infections, improvement of honey bee survival.	[104]
*Eucalyptus globulus*	Ethanolic	essential oils	Reduction of *Nosema* spores.	[99]
*Evernia prunastri*	Ethanolic	phenolic acids and flavonoids (chlorogenic acid, vanilic acid, vanillin, rosmarinic acid, crisin, o-Cumaric acid and acacetin)	Reduction of *Nosema* spores.	[98]
*Humulus lupulus*	Ethanolic	flavonoids (isoquercitrin, rutin, epicatechin)	Reduction of *Nosema* spores.	[98]
*Laurus nobilis*	Ethanolic	phenolic acids and flavonoids (syringic acid, isoquercitrin, quercetin, kaempferol, rutin, epicatechin, resveratrol and monoterpenes (1,8-cineole, sabinene and linalool)	Reduction of *Nosema* spores.	[97,98,107,108]
*Ocimum basilicum*	Ethanolic	phenylpropanoid and phenylpropene (methyl eugenol, methyl chavicol)	Reduction of *Nosema* spores.	[96]
*Origanum vulgare*	Ethanolic	phenolic acids, flavonoids (isoquercitrin, rosmarinic acid, apigenin, vitexin 2-o-ramnoside, sinapic acid, resveratrol) and essential Oils	Reduction of *Nosema* spores.	[98,109]
*Plantago lanceolata*	Aqueous	flavonoids, alkaloids, terpenoids, phenolic compounds (caffeic acid derivatives), fatty acids, polysaccharides	Antimicrobial, antioxidant and cytotoxic activity; reduction of *Nosema* spores; improvement of honey bee survival.	[97]
*Psidium guajava*	Ethanolic	terpenes (limonene, β-Pinene, α-Pinene, caryophyllene)	Reduction of *Nosema* spores.	[96]
*Rosmarinus officinalis*	Aqueous	phenolic acid, terpenes and terpeinods (rosmarinic acid, caffeic acid, ursolic acid, betulinic acid, carnosic acid and carnosol, camphor, 1,8-cineole, α-pinene, borneol, camphene, β-pinene and limonene)	Antimicrobial and antioxidant activity, reduction of *Nosema* spores; improvement of honey bee survival.	[97]
*Rosmarinus officinalis*	Hydroalcoholic	essential oils	Reduction of *Nosema* spores.	[109]
*Rumex acetosella*	Aqueous	phenolic compounds and inorganic salt derivates (tannic acid, binoxalate of potassium, and nitrogenous matter)	Reduction of *Nosema* spores and improvement of honey bee survival.	[97]
*Salvia officinalis*	Aqueous	terpenes and terpenoids (cis-thujone, camphor, cineole, humulene, trans-thujone, camphene, pinene, limonene, bornyl acetate and linalool)	Antimicrobial and antioxidant activity, reduction of *Nosema* spores, improvement of honey bee survival.	[97]
*Syzygium jambos*	Ethanolic	phenolic compounds, anthraquinones, and steroids	Reduction of *Nosema* spores.	[96]
*Thymus vulgaris*	Ethanolic	essential oils	Reduction of *Nosema* spores.	[99]
*Thymus vulgaris*	Aqueous	terpenes and terpenoids (geraniol, linalool, gamma-terpineol, carvacrol, thymol and trans-thujan-4-ol/terpinen-4-ol, p-cymene, γ-terpinene and thymol)	Antimicrobial and antioxidant activity, reduction of *Nosema* spores, improvement of honey bee survival.	[97]
*Ugni molinae*	Methanolic	phenolic acids (caffeic acid)	Reduction of *N. ceranae* spores and improvement of honey bee survival.	[95]
*Urtica dioica*	Ethanolic	essential oils	Reduction of *Nosema* spores.	[99]
*Vaccinium myrtillus*	Ethanolic	phenolic acids and flavonoids (chlorogenic acid, syringic acid, ferulic acid, isoquercitrin, quercetin, myricetin, naringenin, kaempferol)	Reduction of *Nosema* spores.	[98]

Pașca et al. [98] reported that integrating the honey bee diet with different plant extracts (*Agastache foeniculum*, *Artemisia absinthium*, *Evernia prunastri*, *Humulus lupulus*, *Laurus nobilis*, *Origanum vulgare*, and *Vaccinium myrtillus*) decreased the number of *Nosema* spores in a similar way to the commercial product Proitofil. The authors hypothesize that the mechanism of action is attributable to the bioactive compounds, such as phenolic acids and flavonoids contained in these extracts.

Nanetti et al. [104] found that the administration of *Brassica nigra* defatted seed meal in the diet of honey bees reduced the mortality of bees affected by *N. ceranae* spores and increased insect lifespan. *Laurus nobilis* extract (essential oil, hydrolate and its main component) did not cause lethal effects on adult honey bees and significantly inhibited *N. ceranae* development [107,108]. Other studies reported that extracts of *Andrographis paniculata*, *Origanum vulgare*, *Rosmarinus officinalis*, and *Artemisia absinthium* were significantly effective in reducing the number of spores and controlling *Nosema* [100,101,109].

In some studies, the anti-microsporidian activity is related to specific compounds (e.g., phenolic compounds, terpenes, aromatic organic chemical compounds, polysaccharides) contained in the vegetal extracts used [91,96,110,111].

In particular, Mura et al. [91] showed that propolis extracts, containing mainly phenolic acids and flavonoids (caffeic acid, ferulic acid, ellagic acid and quercetin), increase the longevity of bees infected with *N. ceranae* and significantly lower the spore load. Promising results were also observed with chitosan, peptidoglycan and algal polysaccharides. These natural products promote antimicrobial activity and have been shown to stimulate the immune system, thus reducing *N. apis* infection in *A. mellifera* [95,112,113,114,115].

Klassen et al. [116] assessed the effect of the prebiotics eugenol, chitosan and naringenin and the probiotic Protexin^®^ (*Enterococcus faecium*) on *N. ceranae* infection, colony population, honey production and winter survival using field colonies. In spring, treatments with eugenol, Protexin^®^ and naringenin significantly decreased *N. ceranae* infections, increased adult bee populations and increased honey production, whilst chitosan was ineffective.

Ptaszyńska et al. [117] demonstrated the efficacy of porphyrins and biological nitrogen pigments (biochromes). Supplementing the diet with sugar syrup containing these substances showed significant efficacy, preventing the development of microsporidia and decreasing the mortality of infected bees.

Another recent study has shown that the use of acetic and p-coumaric acids in the honey bee diet was effective in the control of nosemosis [111]. The efficacy of p-coumaric acid confirms the results obtained by Bernklau et al. [110], who showed that this substance, together with other phytochemical compounds (caffeine, gallic acid and kaempferol), administered individually, reduces spore load and bee mortality.

The extracts of *Artemisia dubia* and *Aster scaber*, belonging to the Asteraceae family, rich in chlorogenic acid and coumarin, exhibited high potential anti-*Nosema* [102,103]. Instead, Arismendi et al. [95] attribute the significant anti-*Nosema* activity to extracts from leaves of *Aristotelia chilensis* (Elaeocarpaceae) and *Ugni molinae* (Myrtaceae) for their high content of flavonol compounds (rutin and myricetin).

Monoterpenes found in *Cryptocarya alba* (Lauraceae) leaves have reported inhibition activity on *N. ceranae* [105]. Similarly, feeding bees curcumin, a phenolic compound from turmeric (*Curcuma longa,* Zingiberaceae), reduced *Nosema* spp. spore loads and increased the survival of infected bees [118]. Resveratrol (a natural phytoalexin: trans-3,5,4′-trihydroxystilbene) and thymol (terpene; 2-iso propyl-5-methylphenol) appear to be capable of diminishing the level of infection, and thus the mortality of experimentally infected bees [119,120].

In a study conducted by Borges et al. [121], sulforaphane (organosulfur compound) from cruciferous vegetables, carvacrol from *Origanum vulgare* (Lamiaceae) oil and naringenin from citrus have been shown to cause a high reduction in *Nosema* spores.

Tlak Gajger et al. [122] reported in their study that diet supplementation with the pentadecapeptide BPC 157, a well-studied gastrointestinal protective compound, has significant therapeutic effects. Their results showed that this specific oral therapy increased the strength of honey bee colonies, reduced the number of *Nosema* spores and limited midgut lesions of infected honey bees. Based on this scientific evidence, the efficacy of many plant extracts and organic compounds against nosemosis appears to be consolidated. However, the exact mechanism of inhibition by all these compounds is still unclear, and in some cases, the antimicrobial activity of plant extracts is not likely due to a single compound but rather to all of the constituents [102].

Several studies suggested that some phenolic compounds, typical secondary metabolites of many plants, can permeate the cell wall and plasma membrane of spores, destroy the plasma membrane and prevent germination of the spore [91,117]. Another recent study reports that the anti-nosemosis activity of several phenolic and monoterpene compounds is related to the inhibition of the expression of the *N. ceranae* virulence factor encoding the polar tube protein 3 (ptp3) mRNA of the ptp3 gene [123]. Furthermore, some compounds, such as polysaccharides, could have the potential to prevent spore adherence to host cells by producing a thin coating on bees’ ventricular walls [115]. The phytoderivative Nozevit, a preparation that includes polyphenols, vitamins, minerals and amino acids, induces the production of mucous from the epithelial layer of treated bees and additionally coats the peritrophic membrane to form a firm and resilient envelope [85]. Other studies have shown that plant extracts support immunity and improve bees’ resistance to nosemosis. In this regard, Ptaszyńska et al. [106] used *Eleutherococcus senticosus*, belonging to the *Araliaceae* family and commonly known as Siberian ginseng. The extract of this plant, containing eleutheroside B, eleutheroside E and naringenin, proved effective both as a cure and in the prophylaxis of nosemosis. These adaptogenic compounds were important for supporting immunity and improving the resistance of honey bees. 

Although they are not obtained from plants, we report the results obtained upon the application of some extracts from basidiomycetes fungi (Kingdom Fungi) and insects (Kingdom Animalia). Anti-*Nosema* and immune-protective effects of *Agaricus bisporus* and *Agaricus blazei* (Agaricaceae) were observed without any side effects but with immunostimulatory activity in the preventive application.

In two studies conducted by Glavinic et al. [124], the extract of these mushrooms stimulated the expression of abaecin, hymenoptaecin, apidaecin and vitellogenin genes reducing the oxidative stress caused by *N. ceranae* and consequently reducing *N. ceranae* infection [124,125].

Recently, a particular survey was performed by Kunat et al. [126] using aqueous extracts of the carton nest produced by the jet-black ant (*Lasius fuliginosus*) in the management of bees infected with *Nosema*. This study showed that the administration of this extract in the honey bee diet greatly influenced the incidence of the disease, inhibiting the germination of *Nosema* spores. *Lasius fuliginosus* is a species of ant belonging to the subfamily Formicinae [127,128].

Based on the scientific evidence mentioned above, supplementing the honey bee diet with natural extracts would offer an alternative therapy for the control of nosemosis and help reduce the overuse of antibiotics in beekeeping.

## 3. RNA Interference

RNA interference (RNAi) is a post-transcriptional process triggered by the introduction of double-stranded RNA (dsRNA) as a tool that limits the transcript level by either suppressing transcription (transcriptional gene silencing [TGS]) or activating a sequence-specific RNA degradation process (post-transcriptional gene silencing [PTGS]/RNA interference [RNAi]) [129,130]. RNA interference (RNAi) is currently being explored for pesticide activity in agriculture and as a potent and specific strategy for controlling infections of parasites and pathogens in insects, including honey bees [131,132,133,134,135,136,137,138]. Several studies evidence that RNAi might be exploited to regulate *Nosema* gene expression within bee hosts [139,140,141].

Kim et al. [142] examined the control of nosemosis caused by *N. ceranae* using RNAi technology. Double-stranded RNA (dsRNA) for RNAi application targeted the mitosome-related genes of *N. ceranae*. Two dsRNAs, specific to *NCER_101456* and *NCER_100157*, showed high inhibitory effects on spore production. *NCER_101456* and *NCER_100157*, as predicted with FNR1 and FNR2, are ferredoxin NADPH+ reductases, which are flavin enzymes that reduce NADP+ by ferredoxin and are involved in electron transport and biodegradation [143,144]. In addition, these dsRNA treatments significantly increased the survival rate of honey bees [142].

Another recent study used RNAi to lower the expression of polar tube protein 3 (ptp3), a protein essential for sporoplasm injection and microsporidian cellular invasion [145]. He et al. [141] explored the therapeutic potential of silencing the sequences of two *N. ceranae* encoded spore wall protein (SWP) genes employing the RNAi-based methodology. This study revealed that the oral ingestion of dsRNAs corresponding to *SWP8* and *SWP12*, used separately or in combination, could lead to a significant reduction in spore load, improved immunity and extended lifespan of *N. ceranae* infected bees.

Previous studies reported that *N. ceranae* infection could comprehensively and persistently suppress the immune system of the honey bee, causing a higher susceptibility to other bee diseases and senescence [39,96].

An interesting aspect of the RNAi response is that dsRNA treatment might not only result in a knockdown of specific gene expression post-transcriptionally, but it may also regulate a signal transduction cascade, useful for reducing the expression of negative regulators of the honey bee immune response [134]. 

Li et al. [146] reported that *nkd* (Naked Cuticle Gene) mRNA levels in adult bees were upregulated by *N. ceranae* infection (and thus, the parasite may use this mechanism to suppress host immune function) and that ingestion of double-stranded RNA (dsRNA) specific to nkd, efficiently silenced the expression of this gene.

Furthermore, it has been demonstrated that RNAi-mediated knockdown of *nkd* transcripts in *Nosema*-infected bees resulted in upregulation of the expression of several immune genes (Abaecin, Apidaecin, Defensin-1, and PGRP-S2), reduction of *Nosema* spore loads and extension of honey bee life span.

RNAi-mediated knockdown of the genes important for *N. ceranae* viability or honey bee immunoregulation may have the potential to control nosemosis. 

Nevertheless, several obstacles should be considered when evaluating the feasibility of RNAi-based bee medications; in fact, oral delivery of dsRNA to honey bees may lower RNAi efficiency and stability, as digestive enzymes and gut pH can rapidly metabolize and alter the drug sequence before delivery to target mRNA [67].

Although many applications of RNAi have been thoroughly researched, no RNAi-based drugs or pesticides have been approved for agricultural use. Off-target and non-specific effects of RNAi are a major concern in agriculture that will likely slow the approval of RNAi-based treatments for apiarian medicine.

The efficiency of RNAi delivery can be influenced by several factors, which can act alone or in combination. Some of the influencing factors include the life stage of the target insect, stability of the target gene, target tissue site and double-stranded RNA (dsRNA) quantity [147]. Therefore, although the results from the use of RNAi-based therapies are very promising for controlling nosemosis infection in honey bees, more research is needed to implement these biomolecular techniques in beekeeping practice. 

## 4. Beneficial Microbes

The gut microbiota plays a key role in the maintenance of honey bee health, contributing to growth and development, immune function and protection against pathogens [148,149,150]. However, the honey bee microbiota is destabilized (dysbiosis) by natural events such as immunosenescence or by various exogenous factors such as climate, diet, nutritional deficiencies, pathogens, pesticides and environmental pollution [151,152,153,154,155,156,157,158]. The functional outcomes of dysbiosis include poor host development, early mortality and increased susceptibility of bees to pathogens [149,152,159,160,161]. Recent studies provide experimental evidence for a link between nosemosis and dysbiosis in the honey bees’ gut [60,162,163,164,165,166,167,168,169,170,171]. Other studies suggested that management strategies based on re-establishing the microbiota are a promising path to restoring or improving the health of honey bees and that probiotics and several bacterial metabolites may participate in the control of nosemosis, other than increase the survival of infected honey bees [172,173,174,175,176]. Table 2 provides a detailed list of the main effects obtained in the biocontrol of *Nosema* spp. through the use of different microbial cultures.

As shown in Table 2, the most commonly used bacteria belong to the group of lactic acid bacteria and specifically to the species related to *Bifidobacterium*, *Enterococcus* and *Pediococcus*. The action of these bacteria is expressed essentially through an antimicrobial action directed against *Nosema* [175,177] or through the stimulation of the immune system of the honey bee [178].

Maggi et al. [179] investigated the impacts of oral administration of organic acids produced by *Lactobacillus johnsonii* CRL1647 (lactic acid, phenyl-lactic acid and acetic acid) and reported a strong spore load reduction in bees. Similarly, De Piano et al. [180] highlighted a relationship between bacterial metabolites and the presence of *N. ceranae* spores, showing a significant decrease after dietary supplementation with *Lactobacillus johnsonii* AJ5.

**Table 2:** Overview of the main effects obtained in the biocontrol of *Nosema* using different microbial cultures.

**Source**	**Microbial Cultures**	**Relevant Reported Effects**	**Ref.**
**Honey bee** **gastrointestinal tract**	*Lactobacillus johnsonii* AJ5 *L. johnsonii* CRL1647	Oral administration of the metabolites produced by *L. johnsonii* (mainly organic acids) supplemented in syrup reduced the intensity of the disease.	[179,180]
	*L. johnsonii* CRL1647	Reduction of *Nosema* spores.	[181]
	*Lactobacillus kunkeei* *		[182]
	*Lactobacillus salivarius* * A3iob		[183]
	*Lactobacillus plantarum* *	The dysbiosis induced by *Nosema* spp. was lessened by the probiotic *L. plantarum*.	[170]
	*Bacillus subtilis* subsp. *Subtilis* Mori2	Reduction of *Nosema* incidence.	[184]
**Honey** **samples**	*B. subtilis*	Surfactin S2, a cyclic lipopeptide produced by *B. subtilis* C4 exhibited statistically significant anti-*Nosema* activity.	[185]
	*Bacillus sp. (PC2)*	Improvement of honey bee survival.	[175]
**Honey bee** **larvae**	*Parasaccharibacter* *apium*	Improvement of honey bee survival.	[175,186]
**Honey bee** **hive**	Multiple strains: *Bifidobacterium* *asteroides* DSM 20431 *Bifidobacterium* *coryneforme* C155 *Bifidobacterium* *indicum* C449 *L. kunkeei* * Dan39 *L. plantarum* * Dan91 *L*. *johnsonii* Dan92	Reduction of *Nosema* spores.	[187]
**Commercial** **probiotic**	Protexin^®^ (*Enterococcus faecium*)	Reduction of *N. ceranae* incidence increased the population of adult bees and increased honey production.	[116,176]
	Bactocell^®^ (*Pediococcus acidilactici*) Levucell SB^®^ (*Saccharomyces boulardii*)	Improvement of honey bee survival.	[175]
	EM^®^ probiotic for bees: Multiple species of LAB and photosynthetic bacteria.	Reduction of *Nosema* spores increased strength of colonies.	[177]
	APIFLORA (Biowet, Poland) lyophilized selected L *actobacillus* strains (Maria Curie-Skłodowska University in Lublin and University of Life Sciences in Lublin, Poland)	Antagonistic effect toward *N. ceranae* and increased bee survival.	Available at: https://biowet.pl/en/produkty/apiflora-2/ accessed on 9 March 2022
	VETAFARM: *Lactobacillus acidophilus* *Lactobacillus delbruekii sub.bulgaricus* *L. plantarum* * L. rhamnosus *B. bifidum* *Enterococcus faecium*	Reduction of *N. ceranae* incidence increased the population of adult bees and increased honey production.	[176]
	*P. acidilactici* (Lallemand SAS Blagnac, France	Regulate genes involved in honey bee development (vitellogenin), immunity (serine protease 40, defensin) and possibly prevent infection by the parasite *N. ceranae.*	[178]

* Taxonomic correspondence: Lactobacillus kunkeei (currently Apilactobacillus kunkeei); Lactobacillus plantarum (currently Lactiplanbacillus plantarum); Lactobacillus salivarius (currently Ligilactobacillus salivarius).

Baffoni et al. [187] reported that probiotic treatment with Lactobacillus and Bifidobacterium strains successfully lowered the presence of Nosema spores in infected honey bees, thus demonstrating the effectiveness of a microorganism-based preventive strategy.

Other studies reported below have shown that other non-LAB bacteria and yeasts may have antagonistic activity against Nosema. For example, Sabaté et al. [184] reported that the endogenous intestinal bacterium Bacillus subtilis subsp. subtilis Mori2 enhanced queen egg-laying, resulting in more bees and reducing the occurrence of nosemosis; furthermore, they demonstrated that a surfactin, a cyclic lipopeptide synthesized by this bacterium, was also shown to reduce the development of N. ceranae, acting either by direct exposure to the purified spores or incorporated into the bee’s digestive tract.

Moreover, Corby-Harris et al. [186] have shown that Parasaccharibacter apium improves honey bee resistance to Nosema. L. kunkeei (currently Apilactobacillus kunkeei) and L. salivarius A3iob (currently Ligilactobacillus salivarius) administered to honey bee colonies reduced Nosema disease. Feeding caged bees, the honey bee gut bacterium L. kunkeei reduced N. ceranae spore loads compared to the control with untreated bees [182]. 

Regarding the antimicrobial activity of the yeasts, Braglia et al. [111] proved that dietary supplementation with Saccharomyces sp. strain KIA1 was effective in the control of nosemosis.

Different commercial probiotic strains have also been tested as an alternative therapy against N. ceranae infections in honey bees. El Khoury et al. [175] demonstrated that administration of sugar syrup containing P. apium (PC1 sp.), Bacillus sp.(PC2 sp.) and two commercial probiotics, Bactocell^®^ (Pediococcus acidilactici) and Levucell SB^®^ (Saccharomyces boulardii) in the diet of honey bees, significant increases the probability of survival after two weeks in both curative and prophylactic treatments. The results reported in this work support that bacteria within the genus Bacillus and the species P. apium have antagonistic activity against Nosema, in accordance with Sabaté et al. [184] and Corby-Harris et al. [186]. The commercial probiotic VETAFARM (L. acidophilus, L. delbruekii sub. bulgaricus, L. plantarum, L. rhamnosus, Bifidobacterium bifidum, Enterococcus faecium), besides reducing N. ceranae proliferation, increased the survivorship of infected honey bees.

Tlack Gajger et al. [177] found that the commercial probiotic EM^®^ PROBIOTIC FOR BEES administration was followed by a significant reduction in the spore count of Nosema spp. in the colonies, and the strength of the colonies increased. The achievements from these aforementioned investigations have shown that some endogenous gut bacteria and commercial probiotics can sometimes have a biologically relevant antagonistic effect on the development of N. ceranae. However, some studies have indicated that arbitrary probiotic treatments may not have beneficial effects on the host [188]. Complementing the diet of honey bees with inadequate probiotics does not prevent the emergence of nosemosis, may de-regulate the insects’ immune system and could significantly increase bee mortality [189]. Andrearczyk et al. [190] noted an increase in Nosema spp. infections in young bees fed with commercial probiotic strains of Lactobacillus sp. and Saccharomyces cerevisiae. As a whole, this suggests that some generic and inappropriate probiotics are not suitable for feeding honey bees.

In a study conducted by Ptaszyńska et al. [189], a significant increase in Nosema spore counts, compared to the A. mellifera infected control, was caused by two probiotic supplements. The former consisted of Lactobacillus casei, L. plantarum, Saccharomyces cerevisiae and Rhodopseudomonas palustris, while the second consisted of L. acidophilus, L. delbrueckii and Bifidobacterium bifidum. Furthermore, probiotic supplementation accelerated the nosemosis incidence. Probably, increased acidity of the honey bees’ gut, which is a consequence of the uncontrolled growth of inadequate LABs, created conditions favorable to faster nosemosis development. In our opinion, based on all the aforementioned studies, it is clear that only the supplementation of the honey bee diet with appropriate probiotics can have a positive impact on nosemosis control by providing a long-lasting strategy to improve overall honey bee health.

## 5. Conclusions

We believe that the use in beekeeping practice of beneficial microbes, plant extracts and RNAi has enormous potential for biocontrol of nosemosis. However, for systematic application, further studies are needed for these techniques to become reliable and effective tools. The antimicrobial activity of plant extract is mainly due to the presence of phenolic compounds and terpenoids, which possess well-known antimicrobial activity. The effect that these substances may have on bee gut microflora and symbiotic LAB, however, is not fully known. Regarding the RNAi-based antiviral effect, the molecular mechanisms have not been thoroughly characterized, and little is known about the optimal RNAi delivery method for treating honey bees at different developmental stages. The use of appropriate probiotics, unlike synthetic or natural chemical compounds, does not adversely affect the balance of the gut microbiota and is also a technique that can help prevent and treat nosemosis as well as positively impact honey bee welfare.

## Data Availability

Not applicable.
